# Les tumeurs desmoides de la paroi thoracique : à propos de 12 cas

**Published:** 2009-11-10

**Authors:** Marouane Lakranbi, Mohamed Smahi, Mehdi Maidi, Mohammed Bouchikh, Yassine Msougar, Yassine Ouadnouni, Hicham Fenan, Abdellah Achir, Mohammed Caidi, Ahmed Alaziz, Abdellatif Benosman

**Affiliations:** 1 Service de chirurgie thoracique CHU Rabat, Maroc

**Keywords:** Tumeur desmoide, Paroi thoracique, Chirurgie thoracique, Maroc

## Abstract

**Introduction:**

Les tumeurs desmoides sont des tumeurs rares des tissus mous qui peuvent être très agressives localement. A travers l’expérience de notre service, nous analyserons les résultats du traitement chirurgical de ces tumeurs.

**Patients et méthodes:**

De 1980 à 2008, 12 patients ont été opérés pour tumeur desmoide de la paroi thoracique. Le diagnostic a été suspecté sur la base des signes cliniques et radiologiques. Aucun patient n’avait un syndrome de Gardner. L’abord chirurgical a été souvent électif à l’aplomb de la tumeur.

**Résultats:**

La résection a été complète dans 11 cas. La confirmation diagnostique a été apportée par l’étude histologique de la pièce opératoire. La durée du suivi post opératoire variait entre 24 et 180 mois. Une patiente était décédée par insuffisance cardiaque et rénale. 7 cas avaient récidivé, et qui ont été traités par simple résection complète dans 5 cas, dont un avait nécessité une greffe myocutanée ; par ailleurs, deux cas ont été traités par résection incomplète associée à une radiothérapie adjuvante.

**Conclusion:**

La chirurgie des tumeurs desmoides de la paroi thoracique doit être aussi large que possible pour diminuer le risque de récidive.

## Introduction

Les tumeurs desmoïdes sont des proliférations des tissus fibroblastiques, infiltrantes qui ne métastasent pas mais qui ont une très forte tendance à récidiver localement [1].

Ce travail a pour but d’évaluer, à travers l’expérience de notre service, les résultats du traitement chirurgical de ces tumeurs et de définir certains facteurs prédictifs de la récidive locale.

## Patients et méthodes

Il s’agit d’une étude rétrospective portant sur 12 cas de tumeurs desmoides de la paroi thoracique colligés dans le service de chirurgie thoracique au CHU de Rabat sur une période de 28 ans entre 1980 et 2008.

Il y avait 7 hommes et 5 femmes avec un age variant entre 17 et 52 ans. Deux patients avaient été opérés dans leurs antécédents pour un autre type de néoplasie thoracique : l’un, avait été opéré par thoracotomie postéro latérale pour un carcinome bronchique (lobectomie supérieure gauche) 13 ans auparavant et l’autre c’était une femme qui avait bénéficié d’une mastectomie droite pour carcinome mammaire six ans auparavant.

Sur le plan clinique, le maître symptôme était la douleur thoracique retrouvée dans 70% des cas alors que dans 30% des cas, les patients étaient venus consulter pour une masse non douloureuse de la paroi thoracique.

Aucun patient n’avait de syndrome de Gardner. Tous nos patients avaient bénéficié d’une radiographie thoracique et d’un scanner thoracique. Il y’ avait six localisations à gauche et six à droite. Dans 10 cas, la tumeur était de siège latéral ; par ailleurs dans deux cas, le siége était antérieure. Chez les deux patients ayant déjà été opérés pour un autre type de néoplasie, la tumeur était localisée en regard de l’ancienne cicatrice de l’incision chirurgicale.

**Figure 1:**
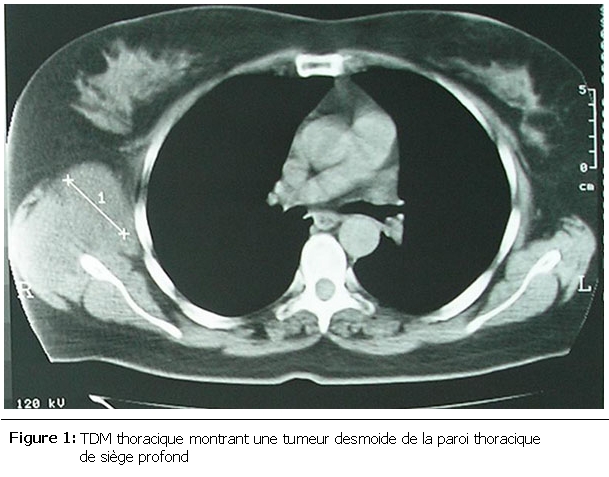
TDM thoracique montrant une tumeur desmoide de la paroi thoracique de siège profond

La taille tumorale variait entre 2 et 13 cm. L’envahissement de la paroi thoracique a été jugé superficiel, limité au plan sous cutané ou au plan musculaire superficiel dans 4 cas, alors que dans 8 cas l’envahissement était profond intéressant le plan musculaire profond et/ou le plan costal ( figure 1 ) et dont un cas s’étendait même en intrathoracique.

La biopsie transpariètale scannoguidée a été faite dans 4 cas mais qui n’était pas concluante mettant en évidence un processus inflammatoire non spécifique. Chez deux patients, le diagnostic de tumeur desmoide a été obtenu en préopératoire par biopsie chirurgicale.

L’abord chirurgical a été électif à l’aplomb de la tumeur dans 11 cas alors qu’un cas avait nécessité une thoracotomie postero-latérale vu que la tumeur se développait surtout en endothoracique. La résection a été complète dans 11 cas et dans un cas, elle a été incomplète avec marges microscopiques positives.

Dans cinq cas, la tumeur adhérait fortement au plan costal et de ce fait une ouverture de la cavité pleurale a été nécessaire afin de réaliser une résection costale plus aisément avec une marge de résection de 5 cm. Aucune résection pulmonaire n’avait été faite. Dans un cas, la perte de substance a été importante nécessitant alors une greffe musculo-cutanée. Dans tous les cas, l’intervention s’était terminée par un drainage du lit tumoral éventuellement associé à un drainage pleural en cas de résection costale.

## Résultats

**Figure 2:**
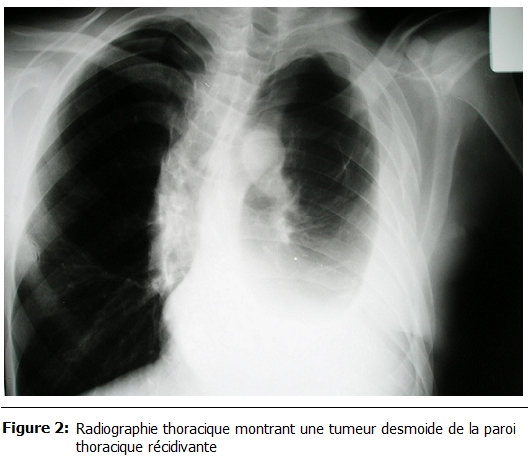
Radiographie thoracique montrant une tumeur desmoide de la paroi thoracique récidivante

La durée d’hospitalisation variait entre 3 et 7 jours soit 4,1 jours en moyenne. Il n’y avait aucun cas de mortalité ni de complications post opératoires immédiates. La durée du suivi post opératoire variait entre 22 et 180 mois. 58% des patients avaient présenté une ou plusieurs récidives (entre une et cinq récidives). Ces récidives avaient été traité à chaque fois par une résection locale seule et dont un cas avait nécessité une greffe d’un lambeau musculo-cutané ; par ailleurs chez deux patients, on avait complété par une radiothérapie adjuvante (50 grays) : l’un avait une tumeur qui s’étendait surtout en intra thoracique avec atteinte du poumon et du médiastin rendant impossible la résection complète ( figures 2 et 3) ; chez ce patient, la croissance tumorale est restée stable avec un recul de deux ans ; l’autre patient, c’était une femme qui avait bénéficié six ans auparavant d’une mastectomie et la radiothérapie adjuvante avait été réalisée chez elle après sa cinquième récidive ; cette patiente était décédée trois mois après par insuffisance cardiaque et rénale. L’ensemble des données sont résumées dans le tableau 1.

## Discussion

Les tumeurs desmodies ont été décrites pour la première fois en 1832 par Mac Farlane [2] ; un siècle plus tard, Mankin soulignait la diversité des localisations. Différentes dénominations ont été attribué à ces tumeurs telles que, les fibromes desmoïdes des parties molles ou fibromes envahissants cependant certains auteurs préfèrent le terme de sarcome de bas grade de malignité [3] vu leur tendance à l’évolution locale ainsi qu’à leur haut risque de récidive même après résection complète.

Toutefois, ces tumeurs fibreuses restent rares, mais non exceptionnelles. En effet, elles représentent approximativement 0,03 % des tumeurs solides [2] et 3,6 % des tumeurs des parties molles avec une incidence de 2 à 4 nouveaux cas par 100000 habitants [4]. L’atteinte concerne sans prédominance raciale, l’adolescent et l’adulte jeune avec une prédilection pour la femme mais reste plus rare chez l’enfant [5]. Dans notre série la prédominance est plutôt masculine.

La localisation thoracique fait partie des tumeurs desmoïdes extra abdominales où elle représente le deuxième site le plus fréquent après l’atteinte des épaules et avant celles des cuisses et de la région cervicale [1] alors que l’atteinte endothoracique reste exceptionnelle [6]. A l’heure actuelle, aucune étiologie n’est certaine, mais trois hypothèses étiopathogéniques occupent le devant de la scène [7] : (1) l’hypothèse traumatique se fonde sur la fréquence relative des tumeurs desmoïdes sur les cicatrices opératoires ; Il s’agirait de la transformation fibreuse d’un hématome [8–9]. (Deux cas dans notre série) ; (2) l’hypothèse hormonale [8–11] part de la constatation d’une croissance rapide de la tumeur chez la femme enceinte et une croissance lente et même des régressions spontanées en période post ménopausique ; (3) l’hypothèse génétique [8–9, 12] est basée d’une part sur l’association fréquente à la polypose colique familiale (syndrome de Gardner), d’autre part sur l’étude de l’arbre généalogique des patients.

La traduction clinique des tumeurs desmoïdes est pauvre. En dehors de la masse qui amène les malades à la consultation, il y a lieu de noter la douleur qui survient quand il y a compression des structures adjacentes en particulier nerveuses. Cette tumeur de découverte souvent fortuite est bosselée, ferme, indolore et n’adhère pratiquement jamais à la peau et se situe en plein corps musculaire [9, 11]. Radiologiquement, c’est une masse des parties molles qui érode souvent le tissu osseux adjacent [13].

Le diagnostic histologique peut être apporté par biopsie chirurgicale permettant d’obtenir de gros fragments alors que la ponction biopsie transparietale est souvent non contributive. Sur le plan microscopique, la lésion est mal limitée avec infiltration des tissus adjacents notamment musculaires.la prolifération est faite de cellules fusiformes séparées les unes des autres par un abondant tissu collagène, sans atypies cellulaires.

Le diagnostic différentiel se pose essentiellement avec un fibrosarcome cependant ce dernier est plus uniformément cellulaire, avec un agencement plus fasciculaire montrant une atypie cellulaire et moins de contingent collagenique, c’est dire l’importance d’une biopsie de gros volume [1]. Le traitement des tumeurs desmoides n’est pas codifié, il comprend plusieurs modalités afin de prévenir le risque de récidive : (1) la chirurgie d’exérèse large de la tumeur si possible tout en respectant les éléments nobles et en acceptant les récidives et les ré-interventions [14]. En cas d’une perte de substance importante, une reconstruction pariétale peut être réalisée en utilisant un matériel prothétique ou un tissu autologue [15–17]; (2) La radiothérapie peut être proposée chez les patients inopérables ou en complément d’une exérèse incomplète pour récidive [18–19] (le cas de nos deux patients); (3) Tous les autres moyens thérapeutiques non chirurgicaux (antimitotiques, anti-estrogènes, modulateur de l’AMP cyclique, inhibiteurs des prostaglandines) n’ont pas encore fait la preuve de leur efficacité mais restent cependant d’intérêt académique et d’avenir [8].

Les récidives surviennent en moyenne dans 50% des cas ; plusieurs facteurs prédictifs de récidive ont été rapportés dans la littérature [1] à savoir : l’âge jeune inférieur à 30 ans, le sexe féminin, la localisation (les récidives sont plus fréquentes au niveau des extrémités), la qualité des marges d’exérèse: le risque de récidive est de 27% si les marges de résection sont histologiquement saines contre 54% si elles sont envahies. Dans notre expérience, nous pensons que le principal facteur prédictif de la récidive est l’envahissement profond de la paroi thoracique ainsi que la positivité des marges de résection.

## Conclusion

La chirurgie reste le traitement de choix des tumeurs desmoides mais des rechutes locales peuvent survenir dans un tiers des cas. Un suivi post opératoire à long terme est nécessaire [20] ; en effet des récidives tardives peuvent survenir même si la résection a été complète et ce probablement à partir d’un nodule de perméation.

## Figures and Tables

**Figure 3:**
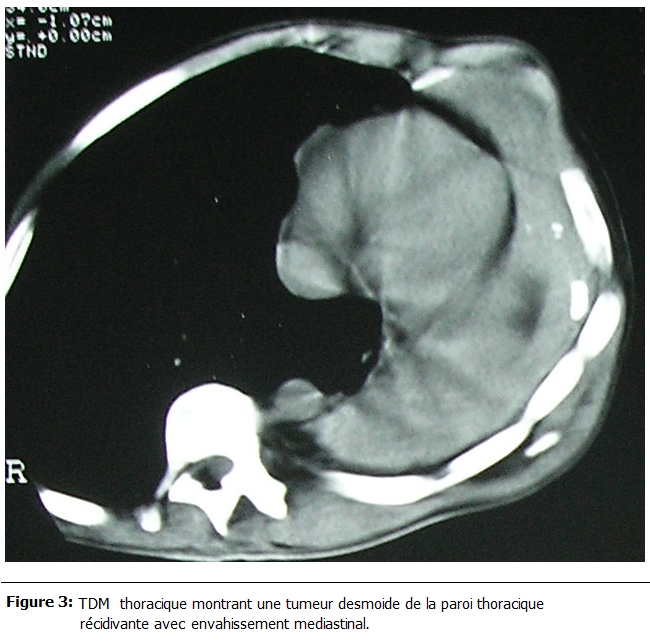
TDM thoracique montrant une tumeur desmoide de la paroi thoracique récidivante avec envahissement mediastinal.
